# Complete genome sequence of plant growth-promoting rhizobacteria strain *Pseudomonas protegens* WSM3457

**DOI:** 10.1128/mra.00749-25

**Published:** 2025-10-29

**Authors:** MacLean G. Kohlmeier, Dinani A. Twite, Graham W. O'Hara, Jason J. Terpolilli

**Affiliations:** 1 Legume Rhizobium Sciences, Food Futures Institute, Murdoch University 5673, Perth, Western Australia, Australia; University of Strathclyde, Glasgow, United Kingdom

**Keywords:** genome sequence, PGPR

## Abstract

We report the complete genome sequence of plant growth-promoting rhizobacteria strain WSM3457. The genome consists of a single 7.1 Mbp chromosome, with phylogenetic and average nucleotide identity comparisons indicating the strain is *Pseudomonas protegens* WSM3457.

## ANNOUNCEMENT

Plant growth-promoting rhizobacteria (PGPR) are bacteria that enhance crop growth and productivity, often by improving rhizobia-legume symbioses (1). Strain WSM3457, isolated from the rhizoplane of annual ryegrass in Swan Valley, Western Australia, in October 2000, was originally named *Alcaligenes xylosoxidans* WSM3457. It demonstrated PGPR activity, increasing the root mass and nodulation of *Trifolium subterraneum* (sub clover) when co-inoculated with *Rhizobium* sp. WSM409 (2). The strain was later renamed *Pseudomonas fluorescens* WSM3457 and shown to enhance nodule initiation and nitrogen accumulation of *Medicago truncatula* (barrel medic) when co-inoculated with *Sinorhizobium medicae* WSM419 (3).

Following isolation, WSM3457 was lyophilized and stored as an ampule in the International Legume Inoculant Genebank at Murdoch University (4). Due to recent taxonomic revisions within *Pseudomonas* (5–7), WSM3457 was selected for complete genome sequencing to support further phylogenetic and functional studies.

An ampule of WSM3457 was opened, revived by resuspension in nutrient broth (Oxoid), and cultured on nutrient agar (Difco) at 28°C. A colony was transferred to nutrient broth and cultured, shaking (250 rpm), at 28°C. This culture was used to produce a −80°C stock and for genomic DNA extraction for short-read sequencing with a Qiagen DNeasy Blood and Tissue Kit. The library was prepared with an NEBNext Ultra II FS DNA Library Prep Kit and sequenced by an Illumina NovaSeq 6000 platform (2×150 bp), generating a total of 4,728,751 paired-end reads. Reads were trimmed for adaptor sequences using bcl2fast v 1.8.4 (Illumina Software) and quality assessed with FastQC v 0.12.1 (8).

For long-read sequencing, the frozen stock was streaked onto LB agar (9) and incubated at 28°C. A colony was grown overnight in LB broth at 28°C, 250 rpm. DNA was extracted using a Qiagen Blood and Cell Culture DNA Mini Kit. The library, prepared with an Oxford Nanopore Technologies Rapid Sequencing Kit SQK-RAD114, was sequenced on a MinION Mk1B with an R10.4.1 flow cell, generating 49,743,646 bp across 29,553 reads (*N*_50_ 12,679). Reads were base called with Dorado v 0.9.2 using the dna_r10.4.1_e8.2_400bps_sup@v4.3.0 model. Unicycler v 0.4.8 (10) used both read sets to assemble one circular contig. The assembly was polished five times with nanopore reads using Racon v 1.5.0 (11) and six times with Illumina reads using Pilon v 1.23 (12). Annotation was completed with the NCBI Prokaryotic Genome Annotation Pipeline v 6.10 (13).

**Fig 1:**
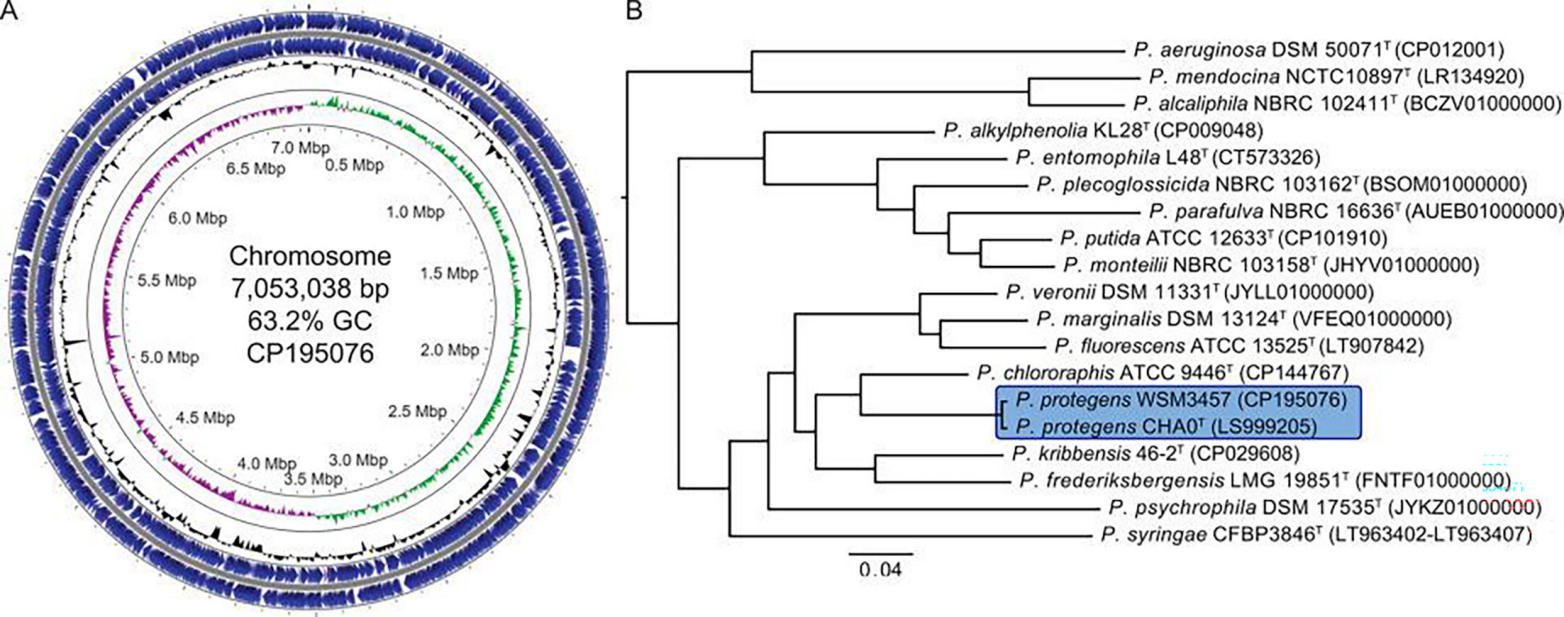
Schematic map of *P. protegens* WSM3457 chromosome (**A**) and phylogenetic relationship to other type strains in the *Pseudomonas* genera (**B**). Inner circles of the map indicate deviations in GC skew (green/purple) and GC content (black), coding sequences are shown in blue. The map was made using Proksee (16) and modified in Adobe Illustrator 2023. The maximum likelihood phylogenetic tree was made from the concatenated and aligned nucleotide sequences of 1,910 core genes, identified using Proteinortho v.6.0.33 (17), with RAxML v.8.2.12 (18) (parameters: *-f a -N 100 -m GTRCAT -T 12*) as previously described (19). The tree was rooted at the midpoint using FigTree v.1.4.4 (https://github.com/rambaut/figtree), the scale bar indicates substitutions per site, and all nodes have 100% bootstrap support. Sequences highlighted in blue have an ANI value of 99.18% as determined by fastANI v.1.33 (20), and accession numbers are added in parentheses.

The final assembly is a single, circular chromosome of 7,053,038 bp with 62.3% GC content, ~208 × coverage, and 99.68% completeness (14) (Fig. 1A). The origin was set to gene *dnaA* using Geneious Prime v 2025.1.3. There are 6,489 predicted genes, including 6,302 protein-coding genes, 16 rRNAs (six 5S, five 16S, and five 23S), 70 tRNAs, four ncRNAs, and 97 pseudogenes. Phylogenetic and Average Nucleotide Identity (ANI) analyses identify WSM3457 as *Pseudomonas protegens*, a species that includes several strains with well-documented beneficial plant root associations (15) (Fig. 1B). The complete genome sequence of *P. protegens* WSM3457 provides a valuable resource for future studies on the strain’s growth-promoting traits.

## Data Availability

This genome sequence project has been deposited in GenBank under BioProject accession number PRJNA1275566. The complete sequence of the chromosome can be found at GenBank under accession number CP195076. Raw Illumina reads and basecalled ONT reads can be found at SRA under accession numbers SRX29144921 and SRX29144922 respectively.
